# Prevalence and trend of multiple coronary artery disease risk factors and their 5-year incidence rate among adult population of Kerman: results from KERCADR study

**DOI:** 10.1186/s12889-023-17504-8

**Published:** 2024-01-02

**Authors:** Nazanin Zeinali-Nezhad, Hamid Najafipour, Mitra Shadkam, Rashed Pourhamidi

**Affiliations:** 1 Physiology Research Center, Institute of Neuropharmacology, Kerman University of Medical Sciences, Kerman, Iran; 2 Cardiovascular Research Center, Institute of Basic and Clinical Physiology Sciences, Kerman University of Medical Sciences, Kerman, Iran; 3 Endocrinology and Metabolism Research Center, Institute of Basic and Clinical Physiology Sciences, Kerman University of Medical Sciences, Kerman, Iran; 4 Non Communicable Diseases Research Center, Bam University of Medical Sciences, Bam, Iran; 5 Department of Physiology and Cardiovascular Research Center, Afzalipour Medical Faculty, Kerman University of Medical Sciences, Kerman, Iran

**Keywords:** Coronary artery disease, Prevalence, Incidence rate, Multiple risk factors, KERCADR study

## Abstract

**Background:**

Coronary artery diseases (CADs) are the most important non‑communicable diseases (NCDs), which cause the highest number of deaths around the world. Hypertension (HTN), dyslipidemia (DL), diabetes mellitus (DM), obesity (OB), low physical activity (LPA), smoking, opium consumption (OC) and anxiety are the most important CAD risk factors, which are more dangerously present in combination in some patients.

**Methods:**

A total of 5835 people aged 15 to 75 years were enrolled in the phase 1 (2012) and followed up to the phase 2 (2017) of the population-based Kerman coronary artery diseases risk factors study (KERCADRS). The prevalence and pattern of different combinations of CAD risk factors (double to quintuple) and their 5-year incidence rates were assessed.

**Results:**

The prevalence of single CAD risk factors (RFs) in phase 2 was 50.2% (DL), 47.1% (LPA), 28.1% (abdominal obesity), 21.2% (OB), 16.5% (HTN), 9.2% (smoking), 9.1% (OC), and 8.4% (DM). The most frequent combination of risk factors was LPA plus DL (23.9%), metabolic syndrome (19.6%), and DL plus OB (17.8%). The 5-year incidence rates of multiple comorbidities (in persons per 100 person-years) was DL plus LPA (2.80%), HTN plus DL (1.53%), and abdominal obesity (AOB) plus DL (1.47%). The most participants (84.4%) suffered from at least one RF, while 54.9% had at least two and 29.9% had at least three RFs.

**Conclusion:**

The results showed that a large portion of the study population suffers from multiple CAD RFs. The findings underscore the importance of identifying multiple CAD risk factors to reduce the overall burden of these NCDs.

## Introduction

Coronary artery diseases (CADs) are a group of cardiovascular diseases (CVDs) that are the most common cause of death all over the world [1–3]. CAD is an atherosclerotic disorder with an inflammatory background that could present in different types such as: stable angina, unstable angina, myocardial infarction (MI), and sudden death [4, 5]. According to the recent studies, CAD risk factors are divided into two main categories, non-modifiable and modifiable risk factors [6, 7]. Age, gender, race, and family history are mentioned as non-modifiable risk factors [8, 9]. Modifiable risk factors include hypertension (HTN), dyslipidemia (DL), diabetes mellitus (DM), obesity (OB), abdominal obesity (AOB), low physical activity (LPA), smoking, opium consumption (OC) and anxiety [10, 11]. The Kerman coronary artery diseases risk factors (KERCADR) study is a population based epidemiological research, which studied 5900 people aged 15–75 years old in phase 1 and 10,000 people in phase 2 in Kerman aimed to investigate the prevalence of CAD risk factors. Recent studies represent strong relation between DM and CAD [12]. As a result, atherosclerotic process happens sooner and is more prevalent in diabetic patients [12–14]. The results of our previous studies demonstrated that the prevalence of HTN, the most common modifiable risk factor, was 19.2% of which 13.9% has already been diagnosed and 5.3% still were unaware of their disease [15]. Also, the relationship between HTN and increased risk of CADs has been proven [16].

DL is a major risk factor for CVDs specially CAD [17, 18]. 56.9% of participants were found to suffer from DL that only 16.2% were already detected and 40.7% were undiscovered [19]. Smoking is classified as one of the modifiable CAD risk factors [20]. Also, long term use of opium is known as a significant risk factor of CAD [21, 22]. On the other hand, OB and overweight can cause CAD [23]. According to KERCADR study phase 1 and 2, the prevalence of OB is 22.3% in phase 2, which increased between the two phases of the study [24, 25]. LPA can intensify the risk of atherosclerosis [26] and it has been estimated that its prevalence is 47.2 in Kerman population and more commonly in women [27].

An Australian study demonstrated that people who had more risk factors were also more likely to report a heart attack, stroke, angina, or atherosclerosis, independent of age and sex [28]. Although only one risk factor is sufficient to increase the risk of a disease, the risk is augmented when another risk factor is added [29]. In a recent cohort study in Shahrekord in southwest Iran, only 39.5 percent of the participants have no non‑communicable diseases (NCD) risk factors; while the people with a co-existence of two, and ≥ 3 NCDs were 16.6 and 15.9 percent respectively [30]. In phase 1 of KERCADRS five years earlier, almost 60% of the people living in Kerman had at least 2 CAD risk factors, and these were more frequent among women and older people [29]. As Iran's predominantly young population approaches middle Ages, there is a growing concern about the burden of CADs in the country in future. To develop effective preventive and management strategies, it is crucial to have accurate and up-to-date information about the trends in the prevalence and incidence of these risk factors, particularly in individuals who have multiple of them. Therefore, the aim of this study was assessing the prevalence of multiple CAD risk factors to find endangered groups of population and comparing the prevalence and assessing the incidence rate of these in a 5-year interval between phases 1 and 2 of KERCADRS. The results can help the health policy makers to have a better insight into the society health status and make plans to decrease the incidence rate of CVDs and the burden of these diseases.

## Methods

### Population and sampling

The first phase of KERCADRS, a population-based, prospective cohort study, was performed in the physiology research center of Kerman from 2009 to 2012 with 5835 participants (having completed data) aged 15 to 75 years old. A total number of 9993 people aged 15 to 80 years old (having completed data) with the mean age of 46.2 ± 15.7 participated in the second phase of the study from 2014 to 2017. The inclusion criteria were: Iranian with at least a 1-year period of residency in Kerman and signing the informed consent form.

The study was conducted in accordance with the principles outlined in the Declaration of Helsinki and in conformity with the national guidelines for conducting human studies. It was approved by the Ethics Committee of Kerman university of medical science, Kerman, Iran (Ethical code: IR.KMU.AH.REC.1400.21).

Written informed consent was obtained from all subjects. For those who were between 15 and 18 years old, the consent was obtained from both the participant and his/her legal guardian.

A skilled physician examined all participants, took their blood pressure, pulse rates and their medical history. A questionnaire consisted of the history of underlying diseases such as DM, HTN, DL, and others, was also completed by the physician. Other necessary data comprising demographic information, height, weight, history of cigarette smoking, OC, and level of physical activity were collected by trained interviewers. 10–12 h fasting blood samples were taken from participants and fasting blood sugar (FBS), total cholesterol, triglyceride (TG), low-density lipoprotein (LDL), and high-density lipoprotein (HDL) were measured using traditional laboratory kits.

### Definition of coronary artery disease risk factors

Participants who had FBS ≥ 126 mg/dL at the time of recruitment or were previously diagnosed with DM or were injecting insulin or taking anti-diabetes medications, were considered as diabetic patients [31]. According to the American heart association (AHA), HTN was defined as a systolic blood pressure ≥ 140 mm Hg or a diastolic blood pressure ≥ 90 mm Hg [32]. Also, people with a history of taking any antihypertensive drug before participating in the study were considered as hypertensive patients.

DL was characterized by total cholesterol ≥ 240 mg/dL or LDL ≥ 160 mg/kg, or HDL < 40 mg/kg in men or HDL < 50 mg/kg in women, or triglyceride (TG) level of ≥ 150 mg/dl, or who were under treatment with lipid-lowering drugs [19].

Physical activity (PA) refers to all activities which were measured by World Health Organization global physical activity questionnaire (GPAQ). Metabolic equivalents of task (METs) were calculated based on the daily activity of the participants to express the intensity of physical activity. MET is the use of energy in an adult while sitting [33]. Low, moderate, and severe physical activity levels were defined based on weekly METs values. A weekly MET value of less than 600 is considered as LPA, while a value between 600 and 3000 is considered moderate. Severe physical activity is defined as a weekly MET value of greater than 3000 [34]. Current cigarette smoking referred to smoke of at least one cigarette per day. OC was referred to regularly consumption of opium by inhalation or orally more than 3 times per week as defined according to the diagnostic and statistical manual of mental disorders-IV (DSM-IV) criteria.

Participant’s height was measured by a tape stadiometer with a minimum measurement of 0.2 cm in a standing position, without shoes, and their weight was measured by a calibrated standard weighing balance (Seca, model 707, Germany). The body mass index (BMI) was calculated through dividing their weight in kilograms by height in meters squared. Participants were categorized as overweight if their BMI was between 25 and 29.9 kg/m^2^ and categorized as obese if their BMI was ≥ 30 kg/m^2^.

Metabolic syndrome is a cluster of conditions that occur together leading to increase the risk of CADs. The syndrome defines as the presence of 3 or more of the criteria of waist circumference ≥ 102 cm in men and ≥ 88 cm in women, TG > 150, HDL < 40 in men and < 50 in women, blood pressure > 130/85 or taking medication for HTN, FBS > 100 mg/dl or taking medications for hyperglycemia [35, 36].

### Statistical analysis

All analyzes were performed using SPSS version 14.1, and *P* < 0.05 was considered as statistically significant. Multiple CADRFs included a combination of two or more factors of HTN, DL, DM, OB, AOB, LPA, smoking, and OC. The reference population was Kerman census population data in 2016. All prevalence rates were presented as percentage frequencies and compared using the chi-squared test among sexes and age groups.

### Incidence rate of multiple CAD risk factors

**Fig. 1:**
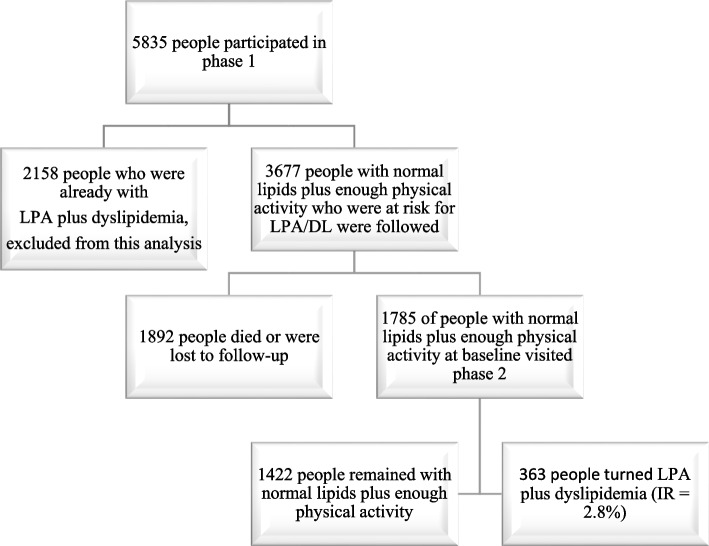
The flow chart of the study for those who participated in both phases of the study (here for calculation of DL plus LPA). *DL* Dyslipidemia, *IR* Incidence rate, *LPA* Low physical activity

For multiple CAD incidence rate calculation those people who participated in phase 1 and followed to phase 2 were considered. Here as an example the incidence rate calculation for LPA plus DL is presented. Out of 5835 participants with complete data in phase 1, 2158 individuals who were already with LPA plus DL, excluded from this analysis. The remaining 3677 participants were physically active enough and had normal lipid levels (were at risk for becoming LPA plus DL) during follow-up. During the follow-up period, 1892 participants died or were lost to follow-up, while 1785 participants (with normal lipids and enough physical activity at baseline) visited phase 2. Of these, 363 individuals developed LPA and DL, yielding an incidence rate of 2.8 per 100 person-years (Fig. 1). To calculate the person-years at risk for those who did not have any CAD risk factors (CADRFs) at the phase 1 visit, the time difference (in years) between the phase 1 and phase 2 visits was calculated. For those lost to follow-up, an average of 2.5 years (half of the overall follow-up time) was assumed to be the person at risk [37].

Using the above information, the incidence rate (expressed as person per 100 person-years) was calculated using the following formula [37]:

$$incidence=\frac{Number\;of\;new\;cases\;of\;LPA\;plus\;DL\;during\;5\;years\;followup}{\left(lost\;to\;follow\;up\;people\;\times2.5\right)+\left(people\;remained\;normal\;between\;phases\;1\&2\;\times\;Time\right)}$$

where Time is the exact duration (in years) between the two attending of each participant in phases 1 and 2.

## Results

### Prevalence of multiple coronary artery disease risk factors

Totally 9993 people were enrolled in phase 2 study of which 4055 (40.6%) were male. The mean age of the participants at enrollment was 46.2 ± 15.7 years. 4669 (46.7%) were jobless or housewife while the other participants had governmental or non-governmental jobs.

The prevalence of DM, HTN, DL, OC, and metabolic syndrome significantly decreased in phase 2 compared with the phase 1 (5 years earlier). The prevalence of OB, AOB, LPA and cigarette smoking increased (Table 1).

**Table 1:** prevalence of coronary artery disease risk factors in phase 1 and 2 of KERCADRS

**Risk Factors**	**Prevalence in phase 1 **			**Prevalence in phase 2**		
	**Total **	**Male**	**Female **	**Total**	**Male**	**Female**
Diabetes mellitus (DM)	9%	8.50%	10.40%	8.40%	6.70%	10%
Hypertension (HTN)	18.40%	20.40%	18.80%	16.50%	16.30%	16.70%
Dyslipidemia (DL)	81.40%	75.80%	87.20%	50.20%	43.80%	56.90%
Obesity (OB)	13%	9.50%	20.10%	21.20%	15.90%	26.60%
Abdominal obesity (AOB)	14.40%	8.20%	26.10%	28.10%	15.40%	41.30%
Smoking	8.30%	15.50%	0.80%	9.20%	17.60%	0.50%
Opium consumption (OC)	10.60%	17.80%	3.60%	9.10%	14.70%	3.30%
Low physical activity (LPA)	42.10%	40.60%	46.40%	47.10%	46.30%	48%
Metabolic syndrome	27.70%	26.50%	29.10%	19.60%	15.40%	23.90%

The prevalence of type 2 diabetes was 6.7% in men that was lower than women (10%). The prevalence of HTN in phase 2 was not statistically different between females (16.7%) and males (16.3%) *(P* = 0.571*).* The prevalence of DL was significantly higher in women (56.9%) than men (43.8). About 26.6% of female participants were obese while the prevalence of OB was lower in men (15.9%). Also, AOB was more frequent in women (41.3%) than men (15.4%). Moreover, the prevalence of cigarette smoking, and OC was more frequent in men than women *(P* < 0.001)*.* The most frequent level of LPA was observed among women and its prevalence was higher (48%) than men (46.3%).

Different combinations of risk factors (two or more simultaneous risk factors) were assessed but the prevalence of most important ones in phases 1 and 2 are reported in Table 2.

**Table 2:** the prevalence of multiple coronary artery disease risk factors in phases 1 and 2 of KERCADRS

**Multiple risk factors**	**Phase 1, n(%)**	**Phase 2, n(%)**	***P*-value**
DM/AOB	359 (2.9)	881(4.6)	< 0.001
DM/OB	226 (1.8)	558(3.1)	< 0.001
DM/DL	792 (7.1)	947(5.4)	< 0.001
DM/smoking	94 (1)	105(0.7)	0.003
DM/LPA	393 (3.4)	784(4.3)	0.178
DM/OC	159 (1.4)	236(1.2)	0.226
DL/AOB	1287 (14.1)	2390(17.8)	< 0.001
DL/OB	1000 (12.3)	1640(13.3)	0.048
DL/smoking	571 (8.6)	437(4.4)	< 0.001
HTN/smoking	109 (1)	218(1.6)	< 0.001
HTN/DL	1238 (10)	1542(9.1)	< 0.001
HTN/OB	405 (3.4)	1065(6.4)	0.045
HTN/AOB	442 (6.3)	1481(8.1)	< 0.001
LPA/OB	519 (6.4)	1307(10.3)	< 0.001
LPA/DL	2380 (37.8)	2579(23.9)	< 0.001
DM/HTN/DL	410 (2.8)	526(2.6)	< 0.001
DM/HTN/smoking	37 (0.2)	49 (0.3)	0.003
DM/LPA/OB	115 (0.9)	305(1.6)	0.002
DM/OC /OB	33 (0.2)	64(0.3)	0.855
DM/DL/OB	222 (1.8)	384(2.2)	0.266
DM/HTN/AOB	217 (1.5)	561(2.7)	< 0.001
DL/smoking/AOB	60 (0.7)	87 (0.8)	< 0.001
LPA/DL/HTN	719(8.3)	776(4.6)	< 0.001
LPA/DL/AOB	583(7.6)	1200(10.1)	< 0.001
LPA/DL/OB	466(6.7)	824(7.1)	0.456
LPA/DL/DM	356(3.7)	513(3.1)	0.019
LPA/DL/Smoking	251(4.3)	223(1.8)	< 0.001
LPA/DL/OC	322(5.3)	319(2.1)	< 0.001
Metabolic syndrome	1621 (27.7)	3087 (19.6)	0.524

*AOB* Abdominal obesity, *DL* Dyslipidemia, *DM* Diabetes mellitus, *HTN* Hypertension, *LPA* Low physical activity. *OB* Obesity, *OC* Opium consumption

The most pravalent double combination risk factor in both phases was LPA/DL (37.8% and 23.9% respectively), which its prevalence was significantly decreased in phase 2 compared with phase 1 (*P* < 0.001). The prevalence of combined DM/AOB and DM/OB increased in phase 2 compared with phase 1. Combination of HTN and OB also appeared to be more frequent in phase 2 (6.4%) than phase 1 (3.4%). Because of significant diminution in the prevalence of DL, the prevalence of DM/DL, smoking/DL, HTN/DL and LPA/DL decreased in phase 2 compared to phase 1. The prevalence of DL/AOB was higher in phase 2 (17.8%) than phase 1 (14.1%). The prevalence of LPA/DM, LPA/OB, LPA/DM/OB, and DM/DL/OB increased in phase 2. Metabolic syndrome appeared to be less frequent in phase 2 than in phase 1 (Table 2).

Due to the existence of more than three CAD risk factors in some of the participants, the prevalence of quadruple and quintuple risk factors in phase 2 are also reported (in clinically important- then in more prevalent combinations order) (Table 3).

**Table 3:** The prevalence of quadruple and quintuple CAD risk factors in participants of the phase 2 of the KERCADR study

Multiple CAD risk factors	Phase 2, n(%)
**Quadruple**	
DM/HTN/AOB/DL	371 (1.8)
DM/HTN/AOB/OB	342 (1.7)
DM/HTN/AOB/LPA	316 (1.5)
DM/HTN/AOB/OC	83 (0.4)
DM/HTN/AOB/Smoking	16 (0.1)
DM/AOB/OB/DL	359 (2.0)
DM/AOB/OB/LPA	286 (1.5)
DM/AOB/OB/OC	59 (0.3)
DM/AOB/OB/Smoking	20 (0.1)
AOB/OB/Smoking/DL	46 (0.5)
AOB/OB/Smoking/LPA	46 (0.5)
AOB/OB/Smoking/OC	27 (0.2)
OB/Smoking/DL/LPA	35 (0.4)
OB/Smoking/DL/OC	21 (0.2)
LPA/DL/OB/AOB	715 (5.3)
LPA/DL/HTN/AOB	483 (2.6)
LPA/DL/HTN/OB	338 (1.9)
LPA/DL/DM/AOB	343 (1.8)
LPA/DL/HTN/DM	289 (1.4)
LPA/DL/DM/OB	207 (1.1)
LPA/DL/Smoking/OC	94 (0.9)
LPA/DL/OC/AOB	115 (0.7)
LPA/DL /OC/HTN	121 (0.7)
LPA/DL/OC/OB	84 (0.6)
LPA/DL/Smoking/AOB	48 (0.5)
LPA/DL/HTN/Smoking	54 (0.4)
LPA/DL/DM/OC	68 (0.4)
LPA/DL/DM/Smoking	32 (0.2)
**Quintuple**	
DM/HTN/AOB/OB/smoking	11 (0.1)
DM/AOB/OB/Smoking/LPA	14 (0.1)
DM/AOB/OB/Smoking/DL	7 (0.1)
DM/AOB/OB/Smoking/OC	7 (0.0)
AOB/OB/Smoking/DL/LPA	26 (0.3)
AOB/OB/Smoking/DL/OC	14 (0.1)
OB/Smoking/DL/LPA/OC	12 (0.2)
LPA/DL/HTN/OB/AOB	310 (1.8)
LPA/DL/HTN/DM/AOB	211 (1)
LPA/DL/HTN/DM/OB	134 (0.6)
LPA/DL/HTN/AOB/OC	64 (0.3)
LPA/DL/HTN/OB/OC	43 (0.2)
LPA/DL/HTN/Smoking/OC	22 (0.2)
LPA/DL/HTN/DM/OC	44 (0.2)
LPA/DL/HTN/DM/Smoking	15 (0.1)
LPA/DL/HTN/AOB/Smoking	17 (0.1)
LPA/DL/HTN/OB/Smoking	10 (0.1)

*AOB* Abdominal obesity, *DL* Dyslipidemia, *DM* Diabetes mellitus, *HTN* Hypertension, *LPA* Low physical activity. *OB* Obesity, *OC* Opium consumption

The prevalence of almost all combined risk factors except those combinations that include smoking and OC, was significantly higher in women in both phases (*P* < 0.001) (Table 4).

**Table 4:** the prevalence of multiple coronary risk factors in phase 1 and 2 of KERCADRS categorized by gender

Risk factors	Prevalence (%)	Male	Female	*P*-value
DM/AOB	phase 1	1.3	4.5	< 0.001
	phase 2	2.1	7.2	< 0.001
DM/OB	phase 1	1.2	2.4	< 0.001
	phase 2	1.7	4.5	< 0.001
HTN/Smoking	phase 1	1.6	0.3	< 0.001
	phase 2	3	0.2	< 0.001
HTN/DL	phase 1	9.7	10.2	0.4
	phase 2	8	10.4	< 0.001
HTN/OB	phase 1	2.5	4.4	< 0.001
	phase 2	4.8	8	< 0.001
HTN/AOB	phase 1	2.7	6.3	< 0.001
	phase 2	5	11.3	< 0.001
DL/AOB	phase 1	7.1	21.3	< 0.001
	phase 2	8.9	27	< 0.001
DL/OB	phase 1	8.7	16	< 0.001
	phase 2	9	17.7	< 0.001
DL/Smoking	phase 1	15.8	1.1	< 0.001
	phase 2	8.4	0.3	< 0.001
DM/DL	phase 1	6	8.3	< 0.001
	phase 2	3.7	7.1	< 0.001
DL/Smoking/AOB	phase 1	1	0.5	0.04
	phase 2	1.5	0.2	< 0.001
DM/HTN/AOB	phase 1	0.7	2.3	< 0.001
	phase 2	1.4	4	< 0.001
DM/HTN/DL	phase 1	2.2	3.4	< 0.001
	phase 2	1.8	3.4	< 0.001
DM/HTN/Smoking	phase 1	0.4	0.1	< 0.001
	phase 2	0.5	0	< 0.001
DM/Smoking	phase 1	1.7	0.2	< 0.001
	phase 2	1.3	0.1	< 0.001
DM/LPA	phase 1	3.1	3.8	0.1
	phase 2	3.4	5.2	< 0.001
DM/OC	phase 1	2.2	0.5	< 0.001
	phase 2	1.7	0.8	< 0.001
LPA/OB	phase 1	4.8	8.1	< 0.001
	phase 2	8.3	12.4	< 0.001
LPA/DL	phase 1	33.2	42.5	< 0.001
	phase 2	21.2	26.7	< 0.001
DM/LPA/OB	phase 1	0.7	1.1	0.08
	phase 2	0.9	2.3	< 0.001
DM/OC/OB	phase 1	0.3	0.1	0.03
	phase 2	0.3	0.3	0.883
DM/DL/OB	phase 1	1.2	2.4	< 0.001
	phase 2	1.1	3.3	< 0.001
DM/OC/LPA	phase 1	0.6	0.7	0.477
	phase 2	0.9	0.4	< 0.001
LPA/DL/ HTN	phase 1	8	8.6	0.347
	phase 2	3.8	5.2	< 0.001
LPA/DL/AOB	phase 1	3.6	11.5	< 0.001
	phase 2	4.4	12.9	< 0.001
LPA/DL/OB	phase 1	4.3	8.8	< 0.001
	phase 2	4.8	8.4	< 0.001
LPA/DL/DM	phase 1	3.1	4.7	< 0.001
	phase 2	2.1	3.7	< 0.001
LPA/DL/OC	phase 1	4.8	5.8	0.126
	phase 2	3.7	1.1	< 0.001
LPA/DL/Smoking	phase 1	3.7	4.7	0.079
	phase 2	4.2	0.2	< 0.001
MetS	phase 1	26.5	29.1	0.012
	phase 2	15.4	23.9	< 0.001

*AOB* Abdominal obesity, *DL* Dyslipidemia, *DM* Diabetes mellitus, *HTN* Hypertension, *LPA* Low physical activity, *MetS* Metabolic syndrom, *OB* Obesity, *OC* Opium consumption

**Fig. 2:**
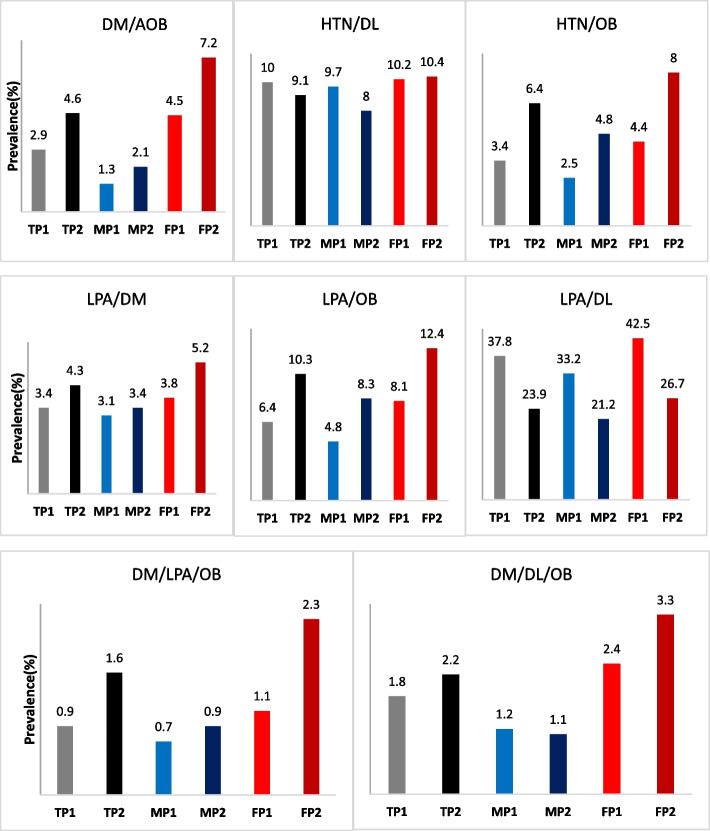
Comparison of the prevalence of multiple CADRFs in phase 1 and 2 of KERCADRS based on gender. In the legend, P1 means phase 1 and P2 means phase 2. TP1, Total phase 1; TP2, Total phase 2; MP1, Male phase1; MP2, Male phase2; FP1, Female phase1; FP2, Female phase2. *AOB* Abdominal obesity, *DL* Dyslipidemia, *DM* Diabetes mellitus, *HTN* Hypertension, *LPA* Low physical activity, *OB* Obesity, *OC* Opium consumption

The prevalence of some of the multiple CAD risk factors increased in the second phase of the study in the gender subcategories. The data shows that 2.1% of men had DM plus AOB in phase 2 which was significantly higher than phase 1 (1.3%). The prevalence of DM plus AOB was 7.2% among women in phase 2, which was 2.7% higher than its prevalence in phase one. Also, the overall prevalence rates of HTN/OB, LPA/DM, LPA/OB and DM/LPA/OB were more frequent in both genders in phase 2 compared to phase 1. Although the combination of HTN/DL was more prevalent among women in phase 2 than in phase 1, its prevalence decreased from 9.7% to 8% among men. The prevalence of LPA/DL was significantly lower in the phase 2 in both genders. complementary information about the prevalence of multiple CAD risk factors in phases 1 and 2 based on gender are shown in Fig. 2.

Furthermore, age plays a significant role in the occurrence of cardiometabolic diseases. The standardized prevalence of multiple CAD risk factors based on differrent age groups are shown in Table 5. The data strongly support that the prevalence of multiple CAD risk factors increases with aging. The most prevalent combination risk factor in young (15–24 years) participants was LPA/DL with 18.7%.

**Table 5:** Standardized prevalence of multiple CAD risk factors among Kerman’s residents based on different age groups

Risk Factors	Standardized prevalence, n (%)						*P* value
	**15–24**	**25–34**	**35–44**	**45–54**	**55–64**	** > 65**	
DM/OB	3(0.3)	11(0.6)	51(2.3)	138(5.9)	238(10.9)	117(9.3)	< 0.001
DM/AOB	4(0.4)	11(0.7)	70(3.1)	210(8.8)	380(17.1)	206(16.3)	< 0.001
DM/LPA	5(0.5)	13(0.8)	57(2.9)	161(7.4)	301(14.1)	247(19.4)	< 0.001
DM/DL	8(0.9)	18(1)	89(4.1)	225(10.2)	390(18.2)	217(17.1)	< 0.001
DM/smoking	0(0)	2(0.1)	9(0.6)	24(1.5)	39(2.3)	31(2.3)	< 0.001
DM/OC	0(0)	0(0)	7(0.4)	35(1.9)	120(6.4)	74(5.7)	< 0.001
DL/LPA	174(18.7)	398(23)	526(26.2)	556(26.5)	564(27)	361(28.5)	< 0.001
DL/OB	56(6.1)	209(11.7)	361(16.5)	421(18.4)	422(19.2)	171(13.5)	< 0.001
DL/AOB	63(6.5)	279(14.7)	495(22)	596(25.3)	645(28.9)	312(24.7)	< 0.001
DL/smoking	8(1)	44(3.2)	89(5.9)	123(8)	126(7.5)	47(3.6)	< 0.001
HTN/smoking	0(0)	7(0.5)	22(1.5)	53(3.4)	72(4.3)	64(4.9)	< 0.001
HTN/DL	2(0.2)	55(3.4)	161(8.1)	352(16.6)	547(25.8)	425(33.2)	< 0.001
HTN/OB	3(0.4)	37(2.3)	118(5.8)	265(11.8)	426(19.8)	216(17)	< 0.001
HTN/AOB	3(0.4)	34(2)	129(6.1)	335(14.5)	588(26.8)	392(30.9)	< 0.001
DM/HTN/AOB	0(0)	3(0.2)	18(0.8)	104(4.4)	268(12.2)	168(13.3)	< 0.001
DM/HTN/DL	0(0)	3(0.2)	22(1.1)	95(4.3)	245(11.3)	161(12.6)	< 0.001
DM/HTN/smoking	0(0)	0(0)	3(0.2)	9(0.6)	15(0.9)	22(1.7)	< 0.001
DM/LPA/OB	2(0.2)	4(0.3)	19(0.9)	63(2.7)	132(6)	85(6.8)	< 0.001
DM/OC/OB	0(0)	0(0)	4(0.2)	5(0.2)	39(2)	16(1.2)	< 0.001
DM/DL/OB	3(0.3)	11(0.6)	39(1.7)	96(4.2)	165(7.4)	70(5.6)	< 0.001
DM/OC/LPA	0(0)	0(0)	7(0.4)	17(1)	56(2.9)	42(3.2)	< 0.001
DL/smoking/AOB	1(0.1)	8(0.6)	20(1.3)	26(1.6)	22(1.2)	10(0.8)	0.002
LPA/DL/HTN	1(0.1)	26(1.7)	61(3.3)	177(8.3)	266(12.4)	245(19.3)	< 0.001
LPA/DL/AOB	38(3.9)	106(5.7)	224(10.2)	280(11.7)	346(15.4)	206(16.5)	< 0.001
LPA/DL/OB	33(3.6)	93(5.4)	154(7.4)	208(8.9)	219(9.8)	117(9.4)	< 0.001
LPA/DL/DM	5(0.5)	8(0.5)	37(1.8)	118(5.3)	208(9.7)	137(10.8)	< 0.001
LPA/DL/smoking	1(0.1)	25(1.8)	48(3.2)	64(4.1)	68(4)	17(1.3)	< 0.001
LPA/DL/OC	0(0)	19(1.4)	39(2.4)	89(5.1)	115(6.2)	57(4.4)	< 0.001
MetS	27 (3)	158 (9.1)	411(19.7)	744 (33.7)	1048(49.4)	699(54.6)	< 0.001

*AOB* Abdominal obesity, *DL* Dyslipidemia, *DM* Diabetes mellitus, *HTN* Hypertension, *LPA* Low physical activity, *MetS* Metabolic syndrom, *OB* Obesity, *OC* Opium consumption

### Incidence rate of multiple coronary artery disease risk factors

The data of 2818 people who participated in both phases 1 and 2 were used for incidence rate (IR) calculation. The IR of multiple CADRFs are summarized in Table 6. The highest incidence rate was observed among the patients who suffered from DL and had LPA (2.80 persons/100 person-years). The IR for HTN plus DL was 1.53 and for DL plus AOB was 1.48 persons/100 person-years, ranking them in the second and third positions.

**Table 6:** The 5-year incidence rate of double and triple coronary artery disease risk factors

Multiple CAD risk factors	Number turned to multiple CAD risk factors	Person- year	Incidence rate (Per 100 person-year)
LPA/DL	363	7283.1	2.81
HTN/DL	242	9321.14	1.53
DL/AOB	248	9966.85	1.48
LPA/OB	273	11,480.32	1.42
HTN/AOB	262	11,278.84	1.40
HTN/OB	231	11,792.47	1.19
DL/OB	199	10,732.04	1.11
DM/LPA	170	12,635.81	0.84
DM/AOB	139	12,850.89	0.68
DL/smoking	118	12,463	0.60
DM/DL	112	11,993.3	0.60
DM/OB	106	13,226.08	0.51
HTN/smoking	86	13,488.09	0.41
DM/ OC	76	13,718.22	0.36
DM/smoking	37	14,021.84	0.17
DM/HTN/AOB	115	13,235.67	0.55
DM/HTN/DL	91	12,842.88	0.45
DM/LPA/OB	73	13,713.07	0.34
DM/DL/OB	68	13,445.91	0.32
DM/LPA/OC	43	14,032.1	0.20
DM/OB/OC	25	14,193.09	0.11
DM/HTN/smoking	24	14,172.02	0.11
DL/AOB/smoking	21	13,906.99	0.10

*AOB* Abdominal obesity, *DL* Dyslipidemia, *DM* Diabetes mellitus, *HTN* Hypertension, *LPA* Low physical activity, *OB* Obesity, *OC* Opium consumption

The results of the distribution of the prevalence of multiple CAD risk factors showed that most of the participants (84.4%) had at least one risk factor of CAD, indicating a high burden of cardiovascular risk in the study population. Moreover, 54.9% had at least two, 29.9% had at least three, and 13.8% had at least four of the studied risk factors. This suggests a high clustering of risk factors in some individuals (Table 7).

**Table 7:** Distribution of the study population according to the prevalence of multiple CAD risk factors

**CAD risk Factors**	**n (%)**	**95% CI**
No risk factor	1076 (15.6)	14.7–16.5
At least one risk factor	8915 (84.4)	83.5–85.3
At least two risk factors	6603 (54.9)	53.8–56.1
At least three risk factors	4108 (29.9)	29–30.9
At least four risk factors	2135 (13.8)	12.8–15

## Discussion

Our study is the first cohort for CADRFs in the southeast of Iran in which we examined the prevalence and trend of multiple CADRFs and their 5-year incidence rate among 15–80-year old population. The study's main result was that the majority of the participants (84.4%), had at least one CADRF leaving only 15.6% without any risk factor. More than half (54.9%) of the participants had two or more risk factors for CAD. In a study conducted by Mamudu et al., in USA, it was found that almost all of their participants (98.3%) had at least one risk factor for developing CAD. Furthermore, 23.5% exhibited two risk factors, 26.4% had three, 19.6% had four, and 14% had five or more risk factors [38]. According to another study conducted by Yamada Harada and colleagues in Japan, the research revealed that the highest proportion of risk factor combination was observed when two risk factors were present. Among individuals without DM, this combination accounted for 39.6% of cases and among those with DM, it accounted for 36.4% of cases [39].

In the present study, the most prevalent combination of multiple risk factors was LPA plus DL (23.9%), followed by metabolic syndrome (19.6%() and DL plus AOB (17.8%). Compared to the first phase of the study, the first two combinations has shown a significant decrease, but the prevalence of DL plus AOB increased. This reduction of first two combination can be attributed to a notable decline in DL between the two phases [19]. However, the increase in the prevalence of AOB has masked the decreasing effect of DL, resulting in an increase in the prevalence of DL plus AOB.

The most prevalent single risk factors are DL, LPA, AOB, and OB which are significant public health concerns. DL was found in half of the study participants (50.2%), LPA in 47.1%, AOB in 28.1% and OB in 21.2% of them.

In the Golestan cohort study in northern Iran, Ahmadi et al. (2016) found that a significant proportion of participants had low levels of physical activity (68.2%), which is higher than its prevalence in our study. Also the prevalence of OB was comparatively higher in Golestan study (25.4%) [40]. In the Shahrekord cohort study in Southwest Iran, also a higher prevalence of several risk factors of CAD was found. These included DM (9.8%), OB (27.1%), LPA (57%), and HTN (17.1%). Additionally, the study found that opium use and smoking were more prevalent in Shahrekord as 12.5% and 24.7%, respectively [30].

In the Azar cohort study, conducted by Farhang et al. in 2019 a higher prevalence of OB, (31.4%), DM (13.9%) and HTN (19.6%) was also found [41]. In an Iranian.

national survey in 2016 a higher prevalence of LPA (51.3%), HTN (25%), smoking (14.2%) was Found. These differences may be due to the age range of the population studied or the regional variations in lifestyle and environmental exposures [42–45]. In the Golestan, Shahrekord and Azar cohorts the population under the studies were in the age of 35 years or higher up to 80 years, which could be one of the reasons for higher prevalence of mentioned CADRFs in their studied population. These findings highlight the significant burden of cardiovascular risk factors in the country and emphasize the need for effective prevention and management strategies nationaly. Fortunately, DL and multiple RFs including DL showed reduction in the prevalence in phase 2 compared to phase 1 of the present study. The reason for reduction of DL in the 5 years between the two phases may be due to the provision of more treatment facilities by the government during this period In Iran [34, 46]. In recent years, the government has performed a health promotion plan that includes covering the treatment costs by providing more efficient health insurance to almost all Iranians. The tendency for replacement of saturated fats with unsaturated oil in cooking may also have played a role. Despite the control of laboratory factors such as blood sugar and plasma lipids, there is still a need for more follow-up for appropriate methods in primary prevention in terms of factors related to physical fitness (including BMI and accumulated body fat level) and lifestyle.

The trends in the prevalence of CAD risk factors over a 5-year period indicate a significant increase in the prevalence of OB, AOB, and low physical activity in the community. Consequently, the combined factors including these risk factors, have also exhibited a noticeable increase in the prevalence. This emphesizes an overall rise in health risks associated with sedentary lifestyles and unhealthy dietary habits [25, 34].

The high incidence rate of multiple CADFRs among the participants of the present study is also of great concern. Among the double clusters LPA/DL with 2.81, HTN/DL with 1.53, and DL/OB with 1.48 person/hundred person-years, and among the triple clusters, DM/HTN/OB with 0.55, DM/HTN/DL with 0.45 and DM/DL/OB with 0.32 person/hundred person-years, have the fastest incidence rate in our studied population. These are probably due to urbanization, sedentary jobs and lifestyle and shortage of time and motivation for having more physical activity [47]. Carrying excess weight in obese subjects can significantly increase the risk of developing mobility issues, such as joint pain and reduced flexibility, which can make it challenging for individuals to engage in physical activity [48]. Furthermore, OB is frequently linked with low self-esteem and body image concerns, which may discourage individuals from participating in physical activity due to fear of judgment or discomfort [49]. As a result, addressing OB and LPA through appropriate lifestyle changes, such as healthy eating habits and regular exercise, can help improve mobility, increase self-esteem, and promote a more active lifestyle. Otherwise the co-occurrence of multiple risk factors increases the likelihood of developing CAD and its associated complications, such as heart attack and stroke.

### Study limitations

The KERCADR study has several limitations that should be considered when interpreting the results. First, the study included only urban population in Kerman, and the results may not be generalizable to rural population. Second, the study relied on self-reported data for some risk factors, such as smoking, opium consumption and physical activity, which may be subject to recall bias, under or over reporting.

## Conclusion

Overall, more than half of the study population have at least two and about one thierd have at least three CAD risk factors. These are valuable information for policymakers and healthcare providers to develop and implement effective strategies for reducing the burden of CADs in Kerman. The high prevalence of CAD risk factors and the clustering of them in mojority of individuals highlight the urgent need for effective preventive strategies, early detection, and management of these risk factors. The decreasing trend in DL and opium consumption prevalences is encouraging, but more efforts are needed to decrease the prevalence and incidence rate of more important metabolic risk factors, such as HTN, DM, OB, AOB, and LPA.

## Data Availability

The datasets used and/or analysed during the present study are available from the corresponding author on reasonable request.
